# Decreased levels of soluble receptor for advanced glycation end products in patients with rheumatoid arthritis indicating deficient inflammatory control

**DOI:** 10.1186/ar1749

**Published:** 2005-04-25

**Authors:** Rille Pullerits, Maria Bokarewa, Leif Dahlberg, Andrej Tarkowski

**Affiliations:** 1 Department of Rheumatology and Inflammation Research, University of Göteborg, Göteborg, Sweden; 2 Joint and Soft Tissue Unit, Department of Clinical Sciences, Lund University, Department of Orthopaedics, Malmö University Hospital, Malmö, Sweden

## Abstract

The receptor for advanced glycation end products (RAGE) is a member of the immunoglobulin superfamily being expressed as a cell surface molecule and binding a variety of ligands. One of these ligands is high-mobility group box chromosomal protein 1, a potent proinflammatory cytokine, expression of which is increased in synovial tissue and in synovial fluid of rheumatoid arthritis (RA) patients. The interaction of high-mobility group box chromosomal protein 1 with cell-surface RAGE leads to an inflammatory response. In contrast, the presence of soluble RAGE (sRAGE) may abrogate cellular activation since the ligand is bound prior to interaction with the surface receptor.

Our aim was to analyse to what extent sRAGE is present in patients with chronic joint inflammation (RA) as compared with patients with non-inflammatory joint disease and with healthy subjects, and to assess whether there is an association between sRAGE levels and disease characteristics.

Matching samples of blood and synovial fluid were collected from 62 patients with RA with acute joint effusion. Blood from 45 healthy individuals, synovial fluid samples from 33 patients with non-inflammatory joint diseases and blood from six patients with non-inflammatory joint diseases were used for comparison. sRAGE levels were analysed using an ELISA.

RA patients displayed significantly decreased blood levels of sRAGE (871 ± 66 pg/ml, *P *< 0.0001) as compared with healthy controls (1290 ± 78 pg/ml) and with patients with non-inflammatory joint disease (1569 ± 168 pg/ml). Importantly, sRAGE levels in the synovial fluid of RA patients (379 ± 36 pg/ml) were lower than in corresponding blood samples and correlated significantly with blood sRAGE. Interestingly, a significantly higher sRAGE level was found in synovial fluid of RA patients treated with methotrexate as compared with patients without disease-modifying anti-rheumatic treatment.

We conclude that a decreased level of sRAGE in patients with RA might increase the propensity towards inflammation, whereas treatment with methotrexate counteracts this feature.

## Introduction

Rheumatoid arthritis (RA) is a chronic inflammatory synovitis that is dominated by the presence of macrophages, lymphocytes and synovial fibroblasts, which leads to the destruction of bone and cartilage. The pathogenesis of the disease is complex, involving a wide range of molecules.

The receptor for advanced glycation end products (RAGE) is a multi-ligand member of the immunoglobulin superfamily being expressed as a cell surface molecule and interacting with a diverse class of ligands [1,2]. RAGE is expressed by many of the cells that participate in the development of RA, including macrophages, neutrophils and T cells. RAGE is expressed on macrophages and T cells within synovial tissues of RA patients as well as on synovial fluid macrophages [3]. Moreover, synovial fibroblasts that account for about 50% of the cellular constituents of the synovial lining layer constitutively express RAGE [4].

The RAGE protein is composed of three immunoglobulin-like regions, a transmembrane domain and a highly charged short cytosolic tail that is essential for post-RAGE signalling. One of the features of the receptor is its recognition of families of ligands, rather than a single protein. The RAGE repertoire of ligands includes products of non-enzymatic glycoxidation (advanced glycation end products), the amyloid-β protein, the S100/calgranulin family of proinflammatory cytokine-like mediators, β2-integrin Mac-1 on leukocytes and the high mobility group box chromosomal protein 1 (HMGB1), all of which are associated with inflammation [2]. Studies have shown that engagement of RAGE by a ligand results in a rapid and sustained cellular activation and gene transcription [1]. Sustained receptor expression leads to a positive feedback loop in which the ligand–receptor interaction increases expression of the receptor itself on the cell surface, leading to further amplification of inflammatory response.

Soluble RAGE (sRAGE), a truncated form of the receptor, is composed of only the extracellular ligand-binding domain lacking the cytosolic and transmembrane domains (i.e. the part that transfers a signal into the cell). This soluble form of the receptor has the same ligand binding specificity and therefore competes with cell-bound RAGE for ligand binding, therefore functioning as a 'decoy' abrogating cellular activation, since the cell surface receptor remains unoccupied. Indeed, it has been demonstrated in a number of experimental animal models that treatment of animals with sRAGE prevents cell-bound RAGE signalling. For example, in a mouse model of collagen-induced arthritis, treatment of mice with sRAGE significantly reduced synovial inflammation, as well as cartilage and bone destruction [5].

In humans, sRAGE is produced by alternative splicing of RAGE mRNA [6-8]. In addition, it has also been shown that pericytes and endothelial cells produce and release sRAGE extracellularly, suggesting the presence of a negative feedback mechanism in RAGE signalling [7]. The proportion and production of the soluble form of the endogenous receptor may therefore influence the regulation of RAGE-mediated functions in various tissues and inflammatory conditions, including RA.

Since sRAGE acts as a competitive receptor for cellular RAGE, the balance between these two types of receptors might be of importance in the pathogenesis of RA. Our aim was to evaluate the levels of sRAGE in patients with RA and to assess whether there is an association between sRAGE levels and disease characteristics. As a comparison, we analysed sRAGE levels in patients with non-inflammatory joint diseases (NIDs) and in healthy subjects.

## Materials and methods

### Patients and controls

Blood and synovial fluid samples were collected from 62 RA patients (mean age 62 ± 13 years, mean disease duration 10 ± 8 years) who met the American College of Rheumatology criteria for RA [9]. Synovial fluids from 33 patients (mean age 43 ± 18 years) with NID were used as controls. In addition, paired blood samples from six NID patients (mean age 58 ± 12 years) were available for analysis. Patients in the NID group were diagnosed to have the following diseases: osteoarthritis, six patients (two blood samples); anterior cruciate ligament rupture, 21 patients; rupture of meniscus, four patients (three blood samples); and knee joint contusion, two patients (one blood sample). All NID patients were examined by an orthopaedic surgeon and a rheumatologist, and chronic inflammatory joint diseases were excluded.

Blood samples from 45 healthy adults with no history of diabetes mellitus or renal disease (mean age 54 ± 9 years) who underwent routine blood testing at the Sahlgrenska University Hospital as blood donors or volunteered in our laboratories were collected to determine serum sRAGE levels in a healthy population. Thirty-six out of 62 RA patients received disease-modifying anti-rheumatic drugs (DMARDs). Methotrexate predominated and was used by 27 patients, either as a monotherapy (19 patients) or in combination with biological agents (six patients [anti-tumour necrosis factor alpha targeted therapy, five patients; anti-IL-1 therapy, one patient]) or sulphasalazin (two patients). One patient was receiving anti-tumour necrosis factor alpha targeted agent in combination with azathioprin and cyclosporin A, while eight patients received monotherapy with other DMARDs (parenteral or oral gold salt compounds, three patients; cyclosporin A, one patient; sulphasalazin, three patients; leflunomide, one patient). The remaining 26 patients, receiving non-steroidal anti-inflammatory drugs or on monotherapy with corticosteroids, were considered as having no DMARD treatment.

The clinical investigation was approved by the Ethical Committee of Göteborg University, and informed consent was obtained from all patients.

### Clinical and laboratory assessment

Clinical examinations were performed by the rheumatologist in all RA patients, and disease activity variables were recorded. The serum concentration of C-reactive protein was measured with a standard nephelometric assay, with normal range 0–5 mg/l. White blood cell counts in the blood were assessed using a microcell counter (F300; Sysmex, Norderstedt, Germany). The white blood cell count in the synovial fluid was also assessed in 24 RA patients.

A murine hybridoma cell line (B13.29, subclone B9), which is dependent on IL-6 for its growth, was employed for the measurement of IL-6 in synovial fluid as previously described in detail [10].

Radiographs of the hands and feet were obtained from all RA patients. Criteria for the erosive disease were the presence of one or more bone erosions, defined as loss of cortical definition of the joint and recorded in proximal interphalangeal joints, metacarpophalangeal joints, carpal joints, wrist joints and metatarsophalangeal joints. Thirty-nine patients out of 62 had erosive disease. The presence of rheumatoid factor of any of the immunoglobulin isotypes was considered positive. Thirty-eight patients had seropositive RA.

Data of patients and healthy controls are summarized in Table 1.

### Collections and preparation of patient samples

Synovial fluid was collected from RA patients who attended the Department of Rheumatology at Sahlgrenska University Hospital in Göteborg with acute knee joint effusion. Synovial fluids were aseptically aspirated and immediately transferred into sodium citrate solution (0.129 mol/l, pH 7.4). Blood samples from the same patients were simultaneously obtained from the cubital vein into the sodium citrate containing tubes. Synovial fluid from NID patients who attended the Department of Orthopaedics at Malmö University Hospital in Malmö was obtained by arthrocentesis.

The collected blood and synovial fluid samples were centrifuged at 2000 × *g *for 10 min, aliquoted, and stored at -70°C until use.

### Reagents

The levels of sRAGE in sera and synovial fluid were determined using a specific sandwich ELISA kit (R&D Systems, Minneapolis, MN, USA) according to the manufacturer's protocol. ELISA plates coated with mouse monoclonal antibody against RAGE were used for quantitative detection of sRAGE. After incubation with blood or synovial fluids, polyclonal capture antibody against the extracellular portion of RAGE was used. The minimum detectable dose of sRAGE was 4 pg/ml. According to the manufacturer, no significant cross-reactivities to EN-RAGE, HMGB1, S100A10 or S100Baa were observed.

Recombinant human HMGB1 was purchased from Sigma (St Louis, MO, USA).

### Statistical analysis

Non-parametric methods were used for statistical comparisons since data showed a non-normal distribution. Statistical differences with respect to sRAGE levels between independent groups were calculated using the Kruskall–Wallis test followed by the Mann–Whitney U test. The Wilcoxon signed rank test for paired samples was used to compare differences between variables in matched samples. Correlations between different variables in patients were assessed with the Spearman rank correlation test. Fisher's exact probability test was used to assess differences between groups with regard to disease characteristics. All sRAGE values are expressed showing the median and the mean ± standard error of the mean. Patients' age and disease duration are reported as the mean ± standard deviation. *P *< 0.05 is considered significant.

## Results

### The levels of sRAGE in blood and synovial fluid

We investigated sRAGE levels in the synovial fluid and in the bloodstream of 62 patients who had RA. Blood samples from 45 healthy controls, synovial fluid from 33 patients with NID and paired blood samples from six patients with NID were assessed as controls.

**Figure 1:**
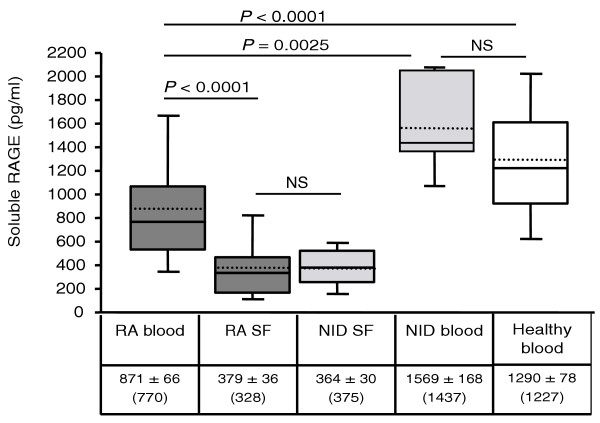
Levels of soluble receptor for advanced glycation end products (soluble RAGE) in blood and synovial fluid (SF) of rheumatoid arthritis (RA) patients and in patients with degenerative/traumatic joint diseases (non-inflammatory joint disease [NID]). In addition, blood levels of soluble RAGE were assessed in healthy controls. Box plots show the 25th and 75th percentiles. Horizontal lines in bold within boxes indicate medians, and dashed lines indicate means. Vertical bars indicate the 5th and 95th percentiles. Statistical differences with respect to soluble RAGE levels between groups were calculated using the Mann–Whitney U test, and differences between paired samples were calculated by the Wilcoxon signed rank test. Mean ± standard error of the mean (median) values are shown. NS, not significant.

**Figure 2:**
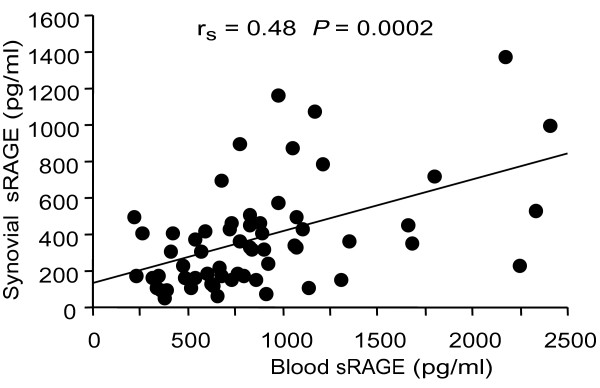
Scattergram showing an association between blood and synovial soluble receptor for advanced glycation end products (sRAGE) levels in rheumatoid arthritis patients. The Spearman rank correlation coefficient (*r*_s_) and *P *value are given.

RA patients displayed significantly decreased (*P *< 0.0001) blood levels of sRAGE (872 ± 65 pg/ml) as compared with healthy controls (1290 ± 78 pg/ml) and with NID patients (1569 ± 168 pg/ml). The sRAGE levels in synovial fluid of RA patients (379 ± 36 pg/ml) were two times lower than in corresponding blood samples (*P *< 0.0001), and were in the same range as in the synovial fluid of patients with NID (364 ± 30 pg/ml) (Fig. 1). There was a significant positive correlation between sRAGE levels in the matching samples of blood and synovial fluid (*r*_s _= 0.48, *P *= 0.0002) (Fig. 2).

**Figure 3:**
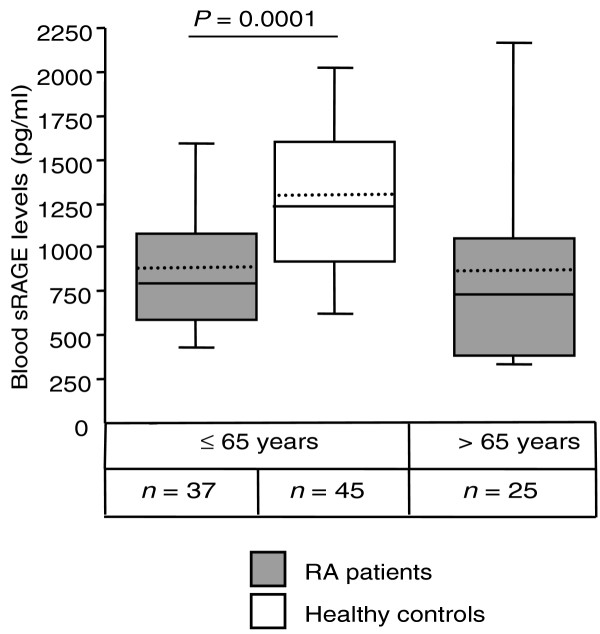
Blood soluble receptor for advanced glycation end products (sRAGE) levels in age-matched groups of rheumatoid arthritis (RA) patients and healthy controls. Box plots show the 25th and 75th percentiles. Horizontal lines within boxes in bold indicate medians, and dashed lines indicate means. Vertical bars indicate the 5th and 95th percentiles. Statistical differences with respect to sRAGE levels between groups were calculated using the Mann–Whitney U test.

Patients who had RA were significantly older than healthy controls and patients with NID (mean age 61.8 ± 13.9 years versus 54.4 ± 9.0 years and 43.0 ± 18.0 years, respectively). However, no correlation with age was found in any of the groups with respect to synovial fluid and blood sRAGE levels. Indeed, when RA patients were stratified into younger (≤ 65 years) and older (>65 years) subgroups, no statistically significant difference was found between these groups with respect to sRAGE levels. Our results indicate, however, that within the age-matched groups (mean age 52.7 ± 10.2 years for RA versus 54.4 ± 9.1 years for controls) up to 65 years of age there was still a major statistical significance regarding circulating sRAGE levels (873 ± 72 pg/ml versus 1290 ± 78 pg/ml, *P *= 0.0001) (Fig. 3). Synovial sRAGE level was in the same range in both younger RA patients (≤ 65 years, 345 ± 36 pg/ml) and in older RA patients (>65 years old, 430 ± 73 pg/ml), and in patients with NID (364 ± 30 pg/ml).

### Correlation between sRAGE levels and clinical features of RA

**Figure 4:**
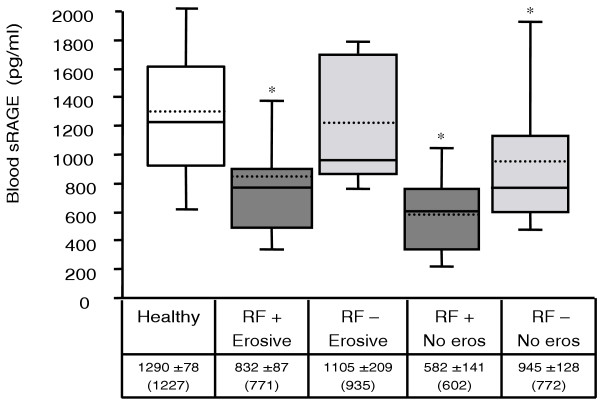
Blood soluble receptor for advanced glycation end products (sRAGE) levels of rheumatoid arthritis patients stratified with respect to seropositivity and erosivity in comparison with healthy controls. Box plots show the 25th and 75th percentiles. Horizontal lines in bold within boxes indicate medians, and dashed lines indicate means. Vertical bars indicate the 5th and 95th percentiles. Statistical differences with respect to sRAGE levels between groups were calculated using the Mann–Whitney U test. The mean ± standard deviation (median) values are shown. * *P *< 0.01 as compared with healthy controls. RF, rheumatoid factor; no eros, no erosion.

We investigated further the association between sRAGE levels with main characteristics of the disease. Stratification of patient data by radiological imaging showed that 39 patients fulfilled the criteria for erosive disease, and 23 patients had no erosions on recent radiographs. There was no difference in patients' age between these two radiographic groups (61.3 ± 12.6 years versus 62.6 ± 16.1 years, respectively). No statistically significant differences in synovial fluid and blood sRAGE levels were found between these two groups (Table 2). However, patients with seropositive RA had a tendency towards lower serum sRAGE levels than patients with seronegative disease (Fig. 4). Blood and synovial levels of sRAGE were not associated with disease duration or acute-phase reactant C-reactive protein. In contrast, the synovial sRAGE levels in RA patients with erosive disease correlated significantly with synovial white blood cell counts (*r*_s _= 0.53, *P *< 0.04), whereas no association was found between synovial fluid sRAGE and synovial IL-6 levels in RA patients.

### The effect of the treatment on sRAGE levels in RA patients

At the time of sampling all patients were receiving anti-inflammatory treatment. Since methotrexate is the most used DMARD in RA treatment and was predominant in our patient population, we decided to investigate whether this treatment had an effect of sRAGE levels in RA patients. A subgroup of patients (*n *= 19) receiving monotherapy with methotrexate was analysed and compared with patients without DMARD treatment (*n *= 26). The patients' data are presented in Table 3.

The baseline characteristics of patients in both groups were similar with respect to age and sex of patients and the presence of rheumatoid factor. However, as expected, patients receiving DMARD treatment had significantly longer disease duration than patients who did not take disease-modifying drugs (13.1 ± 9.6 years versus 8.2 ± 8.3 years, *P *< 0.04). Also, erosive disease was more common in this group (15/19 [79%] versus 10/26 [39%], *P *< 0.02).

**Figure 5:**
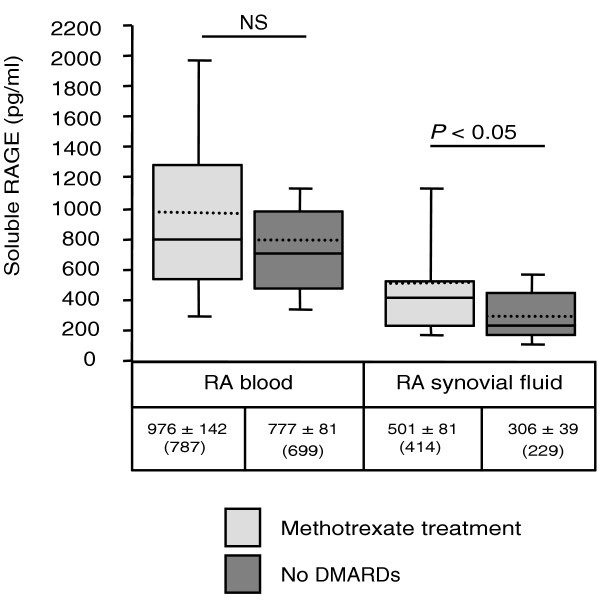
Levels of soluble receptor for advanced glycation end products (soluble RAGE) in blood and synovial fluids of rheumatoid arthritis (RA) patients who received methotrexate treatment or were not treated with disease-modifying antirheumatic drugs (DMARDs) at all. Box plots show the 25th and 75th percentiles. Horizontal lines in bold within boxes indicate medians, and dashed lines indicate means. Vertical bars indicate the 5th and 95th percentiles. Statistical differences with respect to soluble RAGE levels between groups were calculated using the Mann–Whitney U test. Mean ± standard error of the mean (median) values are shown. NS, not significant.

Importantly, significantly higher sRAGE levels were found in the synovial fluid of RA patients treated with methotrexate (Fig. 5) as compared with non-treated patients. Even in this case, the synovial fluid sRAGE displayed significant correlation (*r*_s _= 0.47, *P *< 0.05) with blood levels.

### HMGB1 expression does not influence sRAGE detection by ELISA

One of the high-affinity binding ligands for RAGE is HMGB1. Previous studies have shown that high (microgram) levels of HMGB1 are found in the synovial fluid and sera of RA patients [11,12]. In addition, we demonstrated (results not shown) that blood sRAGE in RA patients may be found on Western blot examination at 60–80 kDa, indicating *in vivo *or *in vitro *complex formation or dimerization. The complex formation between these two proteins could possibly affect the measurement of sRAGE by ELISA.

This prompted us to test whether HMGB1 binding to sRAGE influenced the detection of the latter in our experimental settings. If it were the case, the decreased sRAGE levels found in our RA patient population would be explained by *in vivo *or *ex vivo *HMGB1 interaction. Recombinant human RAGE in concentrations of 500 pg/ml and 2000 pg/ml was incubated with different concentrations (0, 0.1, 1 and 10 μg/ml) of recombinant human HMGB1, and a standard ELISA analysis was performed. Our results showed that HMGB1 did not affect the sRAGE detection by ELISA (data not shown), indicating that lower sRAGE levels measured in RA patients are not due to soluble receptor engagement with HMGB1.

## Discussion

This is the first study examining sRAGE levels in patients with RA. Cell surface RAGE expression is largely dictated by the interaction with its ligands. The expression of cellular RAGE is rather low in mature animals and in human adults. Accumulation of RAGE ligands results in increased expression of the cell surface receptor itself [13]. Furthermore, the receptor–ligand interaction leads to increased RAGE-mediated signalling, resulting in an activation of several intracellular pathways including NF-κB [14].

sRAGE, a truncated form of the receptor, binds ligands with affinity equal to that of cellular RAGE. It therefore has the ability to prevent RAGE signalling acting as a decoy by binding ligands and preventing them from reaching cell surface RAGE. sRAGE has successfully been used in variety of animal disease models to antagonize RAGE-mediated pathologic processes [5,14-16]. Experiments to date have shown that pericytes and endothelial cells produce and release RAGE extracellularly, suggesting the presence of a negative feedback mechanism and immune surveillance mechanisms in RAGE signalling [7].

In our study, we found that RA patients have significantly decreased blood levels of sRAGE as compared with the healthy population and patients with NID. Why do RA patients display low levels of sRAGE? In the case of RA, there is a wide diversity of RAGE ligands present in the inflamed joints, as well as in the circulation, that could lead to the binding and consumption of sRAGE during the inflammatory process. One of the high-affinity ligands for RAGE/sRAGE is HMGB1, a potent cytokine playing an important role in the pathogenesis of chronic inflammation. HMGB1 is a potent trigger of arthritis and its expression is increased in synovial tissue of RA patients as well as in experimental arthritis [12,17]. HMGB1 levels in the synovial fluid and sera of RA patients are significantly elevated as compared with levels in osteoarthritis patients [11,18]. It is thus probable that sRAGE may form *in vivo *complexes with HMGB1 in the sera/synovial fluid of RA patients, leading to inaccurately low levels of sRAGE. Upon co-incubation of these two proteins, however, HMGB1 binding to sRAGE did not affect the detection of the latter, indicating that lower sRAGE levels measured in RA patients are not due to neutralization by HMGB1.

An alternative explanation for the decreased sRAGE levels in RA might be a true consumption of this molecule. In the inflammatory milieu, such as in the rheumatoid joint, other sRAGE ligands also exist. Foell and colleagues have recently reported that extracellular newly identified RAGE-binding protein (EN-RAGE), a member of the S100/calgranulin family, was strongly expressed in inflamed synovial tissue. Furthermore, highly increased serum and synovial fluid levels of EN-RAGE were found in arthritic patients in comparison with control subjects [19]. Finally, raised advanced glycation end product levels have been found in serum and synovial fluid of patients with RA [20]. The presence of high levels of these soluble ligands in RA patients provides a basis for increased consumption of the sRAGE by interaction, followed by elimination of such sRAGE–ligand complexes via the reticuloendothelial system [21].

In addition, cell-bound RAGE functions as a counter-receptor for leukocyte integrins, thereby being directly involved in leukocyte recruitment, especially in inflammatory conditions when the receptor expression increases [22]. Also, in this context, sRAGE has been suggested to function as a potential inhibitor of leukocyte recruitment [22]. In RA patients with erosive disease, we observed a positive correlation between the white blood cell count and synovial sRAGE levels, indicating that endothelial cells in the synovial blood secrete sRAGE extracellularly as a negative feedback mechanism to limit the inflammation. Alternatively, MMP-9 has been found to shed cell-bound RAGE into the culture medium in mice [23]. It is possible that in the rheumatoid joint, where expression of MMP-8 and MMP-9 is increased [24], sRAGE levels are regulated by matrix metalloproteinases in a similar manner.

Taken together, we suggest that soluble RAGE may block the ligand–RAGE interaction on the cell surface by directly binding leukocyte β2-integrin Mac-1 and thereby decreasing influx of inflammatory cells into the joint cavity, functioning as an immune surveillance mechanism. Lower levels of sRAGE detected in RA patients might thus increase the propensity towards inflammation since RAGE ligands have better access to cell membrane-bound receptor, the binding of which leads to the activation of inflammatory pathways.

Consistent with this concept, RA patients treated with methotrexate, one of the most efficient DMARDs, displayed increased sRAGE as compared with RA patients with no immunosuppressive treatment. It is known that methotrexate induces an increase of extracellular adenosine, which further downregulates the expression of adhesion molecules including β2-integrin Mac-1, a ligand for RAGE/sRAGE [25,26]. Methotrexate is also known to downregulate EN-RAGE expression in the synovium of arthritis patients [19] and to suppress activity of tumour necrosis factor alpha [25,27], the cytokine that has been shown to upregulate cellular RAGE [28]. Hypothetically, as the level of membrane-bound receptor and its ligands declines with treatment, less sRAGE is consumed and the balance is restored.

We found that sRAGE levels in RA patients' synovial fluid and sera displayed strong correlation on an individual level. Diverse splicing variants of RAGE have been found in many tissues and the proportion seems to differ between individuals [6-8]. The proportion and production of the soluble form of the endogenous receptor may therefore influence the regulation of RAGE-mediated functions in various tissues and inflammatory conditions, including RA. Whether low sRAGE levels in RA patients are the consequence of the disease or a potential contributing factor to the disease needs to be elucidated.

## Conclusion

We conclude that a decreased level of sRAGE in patients with RA might increase the propensity towards inflammation, whereas treatment with methotrexate counteracts this feature.

## Abbreviations

DMARD = disease-modifying anti-rheumatic drug; ELISA = enzyme-linked immunosorbent assay; EN-RAGE = extracellular newly identified RAGE-binding protein; HMGB1 = high-mobility group box chromosomal protein 1; IL = interleukin; NID = non-inflammatory joint disease; RA = rheumatoid arthritis; RAGE = receptor for advanced glycation end products; sRAGE = soluble receptor for advanced glycation end products.

## Competing interests

The author(s) declare that they have no competing interests.

## Authors' contributions

RP carried out all the experiments, performed the statistical analyses and wrote the manuscript. MB and LD participated in patients' examinations, provided samples from synovial fluid/blood as well as collected clinical data about patient groups. AT conceived of the study, participated in its design and helped in the writing of the manuscript.

## Figures and Tables

**Table 1:** Clinical and demographic characteristics of patients and healthy controls

	Rheumatoid arthritis patients	Non-inflammatory joint disease patients	Healthy controls
Patients	62	33	45
Age (years ± standard deviation)	61.8 ± 13.9	43.0 ± 18.0	54.4 ± 9.0
Sex (male/female)	18/44	20/13	2/43
Disease duration (years ± standard deviation)	10.1 ± 8.5		
Rheumatoid factor (+/-)	38/24		
Radiographic changes (erosive/non-erosive)	39/23		
Treatment (DMARD/no DMARD)	36/26		

DMARD, disease-modifying anti-rheumatic drug.

**Table 2:** Levels of soluble receptor for advanced glycation end products (sRAGE) in sera and in synovial fluid of rheumatoid arthritis patients according to different disease characteristics

Disease characteristic	*n*	Blood sRAGE	Synovial sRAGE
Erosive rheumatoid arthritis	39		
Rheumatoid factor-positive	33	832 ± 87 (771)	345 ± 39 (323)
Rheumatoid factor-negative	6	1105 ± 209 (935)	447 ± 171 (273)
Non-erosive rheumatoid arthritis	23		
Rheumatoid factor-positive	5	582 ± 141 (602)	463 ± 153 (498)
Rheumatoid factor-negative	18	945 ± 128 (772)	397 ± 79 (281)

Data presented as the mean ± standard error of the mean (median).

**Table 3:** Clinical and demographic characteristics of patients receiving disease-modifying anti-rheumatic treatment with methotrexate or having no disease-modifying anti-rheumatic drug (DMARD) treatment

Characteristic	Methotrexate treated	No DMARD
Patients (*n*)	19	26
Age (years ± standard deviation)	63.8 ± 14.3	60.8 ± 14.0
Sex (male/female)	5/14	8/18
Disease duration (years ± standard deviation)	13.1 ± 9.6*	8.2 ± 8.3
Rheumatoid factor (+/-)	12/7	14/12
Radiographic data (erosive/non-erosive)	15/4*	10/16

* *P *< 0.05 as compared with patients without DMARD treatment.

## References

[B1] Schmidt AM, Yan SD, Yan SF, Stern DM (2001). The multiligand receptor RAGE as a progression factor amplifying immune and inflammatory responses. J Clin Invest.

[B2] Schmidt AM, Yan SD, Yan SF, Stern DM (2000). The biology of the receptor for advanced glycation end products and its ligands. Biochim Biophys Acta.

[B3] Drinda S, Franke S, Ruster M, Petrow P, Pullig O, Stein G, Hein G Identification of the receptor for advanced glycation end products in synovial tissue of patients with rheumatoid arthritis. Rheumatol Int.

[B4] Hou FF, Jiang JP, Guo JQ, Wang GB, Zhang X, Stern DM, Schmidt AM, Owen WF (2002). Receptor for advanced glycation end products on human synovial fibroblasts: role in the pathogenesis of dialysis-related amyloidosis. J Am Soc Nephrol.

[B5] Hofmann MA, Drury S, Hudson BI, Gleason MR, Qu W, Lu Y, Lalla E, Chitnis S, Monteiro J, Stickland MH (2002). RAGE and arthritis: the G82S polymorphism amplifies the inflammatory response. Genes Immun.

[B6] Malherbe P, Richards JG, Gaillard H, Thompson A, Diener C, Schuler A, Huber G (1999). cDNA cloning of a novel secreted isoform of the human receptor for advanced glycation end products and characterization of cells co-expressing cell-surface scavenger receptors and Swedish mutant amyloid precursor protein. Brain Res Mol Brain Res.

[B7] Yonekura H, Yamamoto Y, Sakurai S, Petrova RG, Abedin MJ, Li H, Yasui K, Takeuchi M, Makita Z, Takasawa S (2003). Novel splice variants of the receptor for advanced glycation end-products expressed in human vascular endothelial cells and pericytes, and their putative roles in diabetes-induced vascular injury. Biochem J.

[B8] Park IH, Yeon SI, Youn JH, Choi JE, Sasaki N, Choi IH, Shin JS (2004). Expression of a novel secreted splice variant of the receptor for advanced glycation end products (RAGE) in human brain astrocytes and peripheral blood mononuclear cells. Mol Immunol.

[B9] Arnett FC, Edworthy SM, Bloch DA, McShane DJ, Fries JF, Cooper NS, Healey LA, Kaplan SR, Liang MH, Luthra HS (1988). The American Rheumatism Association 1987 revised criteria for the classification of rheumatoid arthritis. Arthritis Rheum.

[B10] Pullerits R, Bokarewa M, Jonsson IM, Verdrengh M, Tarkowski A (2005). Extracellular cytochrome c, a mitochondrial apoptosis-related protein, induces arthritis. Rheumatology (Oxford).

[B11] Taniguchi N, Kawahara K, Yone K, Hashiguchi T, Yamakuchi M, Goto M, Inoue K, Yamada S, Ijiri K, Matsunaga S (2003). High mobility group box chromosomal protein 1 plays a role in the pathogenesis of rheumatoid arthritis as a novel cytokine. Arthritis Rheum.

[B12] Andersson U, Erlandsson-Harris H (2004). HMGB1 is a potent trigger of arthritis. J Intern Med.

[B13] Stern D, Du Yan S, Fang Yan S, Marie Schmidt A (2002). Receptor for advanced glycation endproducts: a multiligand receptor magnifying cell stress in diverse pathologic settings. Adv Drug Deliv Rev.

[B14] Schmidt AM, Hofmann M, Taguchi A, Yan SD, Stern DM (2000). RAGE: a multiligand receptor contributing to the cellular response in diabetic vasculopathy and inflammation. Semin Thromb Hemost.

[B15] Hofmann MA, Drury S, Fu C, Qu W, Taguchi A, Lu Y, Avila C, Kambham N, Bierhaus A, Nawroth P (1999). RAGE mediates a novel proinflammatory axis: a central cell surface receptor for S100/calgranulin polypeptides. Cell.

[B16] Taguchi A, Blood DC, del Toro G, Canet A, Lee DC, Qu W, Tanji N, Lu Y, Lalla E, Fu C (2000). Blockade of RAGE-amphoterin signalling suppresses tumour growth and metastases. Nature.

[B17] Pullerits R, Jonsson IM, Verdrengh M, Bokarewa M, Andersson U, Erlandsson-Harris H, Tarkowski A (2003). High mobility group box chromosomal protein 1, a DNA binding cytokine, induces arthritis. Arthritis Rheum.

[B18] Ulloa L, Batliwalla FM, Andersson U, Gregersen PK, Tracey KJ (2003). High mobility group box chromosomal protein 1 as a nuclear protein, cytokine, and potential therapeutic target in arthritis. Arthritis Rheum.

[B19] Foell D, Kane D, Bresnihan B, Vogl T, Nacken W, Sorg C, Fitzgerald O, Roth J (2003). Expression of the pro-inflammatory protein S100A12 (EN-RAGE) in rheumatoid and psoriatic arthritis. Rheumatology (Oxford).

[B20] Drinda S, Franke S, Canet CC, Petrow P, Brauer R, Huttich C, Stein G, Hein G (2002). Identification of the advanced glycation end products N(epsilon)-carboxymethyllysine in the synovial tissue of patients with rheumatoid arthritis. Ann Rheum Dis.

[B21] Renard C, Chappey O, Wautier MP, Nagashima M, Lundh E, Morser J, Zhao L, Schmidt AM, Scherrmann JM, Wautier JL (1997). Recombinant advanced glycation end product receptor pharmacokinetics in normal and diabetic rats. Mol Pharmacol.

[B22] Chavakis T, Bierhaus A, Al-Fakhri N, Schneider D, Witte S, Linn T, Nagashima M, Morser J, Arnold B, Preissner KT, Nawroth PP (2003). The pattern recognition receptor (RAGE) is a counterreceptor for leukocyte integrins: a novel pathway for inflammatory cell recruitment. J Exp Med.

[B23] Devaux Y, Senior RM, Ray P (2004). RAGE: a new target for MMP-9 in the regulation of inflammatory response in the lung during oxidative stress. Am J Respir Crit Care Med.

[B24] Tchetverikov I, Ronday HK, Van El B, Kiers GH, Verzijl N, TeKoppele JM, Huizinga TW, DeGroot J, Hanemaaijer R (2004). MMP profile in paired serum and synovial fluid samples of patients with rheumatoid arthritis. Ann Rheum Dis.

[B25] Chan ES, Cronstein BN (2002). Molecular action of methotrexate in inflammatory diseases. Arthritis Res.

[B26] Wollner A, Wollner S, Smith JB (1993). Acting via A2 receptors, adenosine inhibits the upregulation of Mac-1 (Cd11b/CD18) expression on FMLP-stimulated neutrophils. Am J Respir Cell Mol Biol.

[B27] Sajjadi FG, Takabayashi K, Foster AC, Domingo RC, Firestein GS (1996). Inhibition of TNF-alpha expression by adenosine: role of A3 adenosine receptors. J Immunol.

[B28] Tanaka N, Yonekura H, Yamagishi S, Fujimori H, Yamamoto Y, Yamamoto H (2000). The receptor for advanced glycation end products is induced by the glycation products themselves and tumor necrosis factor-alpha through nuclear factor-kappa B, and by 17beta-estradiol through Sp-1 in human vascular endothelial cells. J Biol Chem.

